# Graphene Oxide–Antisense miR-21 Nanosystem Modulates Gene Expression and Suppresses Tumorigenesis in HepG2-Derived CAM Xenografts

**DOI:** 10.3390/biom16040523

**Published:** 2026-04-01

**Authors:** Paola Trischitta, Barbara Nasiłowska, Rosamaria Pennisi, Marianna Costa, Maria Teresa Sciortino, Marta Kutwin

**Affiliations:** 1 Department of Chemical, Biological, Pharmaceutical and Environmental Science, University of Messina, Viale Ferdinando Stagno d’Alcontres 31, 98166 Messina, Italy; paola.trischitta@studenti.unime.it (P.T.); rpennisi@unime.it (R.P.); marianna.costa1@studenti.unime.it (M.C.); 2 Department of Chemistry, Biology, and Biotechnology, University of Perugia, Via Elce di Sotto 8, 06123 Perugia, Italy; 3 Institute of Optoelectronics, Military University of Technology, Gen. S. Kaliskiego 2, 00-908 Warsaw, Poland; barbara.nasilowska@wat.edu.pl; 4 Department of Nanobiotechnology, Institute of Biology, Warsaw University of Life Sciences, Ciszewskiego 8, 02-786 Warsaw, Poland

**Keywords:** graphene oxide, miR-21, hepatocellular carcinoma, CAM model, anti-angiogenic therapy, nanocarrier

## Abstract

Graphene oxide (GO) is a promising nanocarrier for the delivery of oligonucleotides. It offers a high loading capacity, efficient cellular uptake, and surface functionalization. MicroRNA-21 (miR-21) is a well-characterized oncomiR commonly overexpressed in hepatocellular carcinoma (HCC). In HCC, miR-21 contributes to tumor progression, inflammation, and angiogenesis. In a previous in vitro study, we showed that GO alone induces the upregulation of pro-inflammatory and tumor-related genes in HepG2 cells. However, conjugation with an antisense miR-21 (GO-antisense miRNA 21) reverses this effect, suggesting a potential therapeutic application. This study aims to evaluate the antitumor and anti-angiogenic efficacy of the GO-antisense miR-21 nanosystem *in ovo* using the chick embryo chorioallantoic membrane (CAM) model. Fertilized chicken eggs (*n* = 4 per group) were randomized into untreated, GO-treated, and GO–antisense miR-21-treated cohorts. A dose of 200 μL (GO 10.0 µg/mL: antisense miR-21 5.0 pmol/mL) was administered intratumorally. Tumor size, volume, and vascularization were monitored through stereomicroscopy and histological analysis. The expression of inflammatory and tumor-associated genes (IL-8, MCP-1, TIMP-2, ICAM-1 and NF-kB) was assessed by quantitative PCR. Given its prominent response, IL-8 protein expression was further analyzed via immunofluorescence. To evaluate tumor-specific delivery, FITC-labeled GO was tracked by confocal microscopy. Our data revealed that treatment with unfunctionalized graphene oxide (GO) unexpectedly promoted tumor vascularization and led to a significant increase in tumor weight. This was accompanied by upregulation of inflammatory markers. In contrast, GO-antisense miR-21 significantly reduced the tumor volume and vessel density. It also successfully downregulated all target genes. Confocal imaging demonstrated preferential accumulation of the nanosystem within the tumor mass. Our results highlight the dual anti-inflammatory and anti-angiogenic effects of GO-antisense miRNA 21 *in ovo* and support its potential as a targeted nanoplatform for HCC treatment.

## 1. Introduction

In recent years, the use of nanomaterials for delivering nucleic acid-based therapeutics has emerged as a promising strategy in oncology. This approach aims to improve target specificity, bioavailability, and intracellular uptake of otherwise unstable molecules [1,2,3]. Among these, graphene oxide (GO) has attracted considerable attention for its unique physicochemical properties. GO has a large surface area and high biocompatibility, and it offers substantial potential for functionalization. It can form stable interactions with oligonucleotides through π–π stacking and hydrogen bonding [4,5]. GO-based nanocarriers have shown potential not only as passive vehicles but also as modulators of gene expression and immune-related pathways [6,7,8,9,10,11].

MicroRNA-21 (miR-21) is consistently upregulated in various human cancers [12], including hepatocellular carcinoma (HCC). In HCC, it acts as an oncomiR by targeting multiple tumor suppressors and promoting proliferation, invasion, angiogenesis, and resistance to apoptosis [13,14,15,16]. As such, antisense oligonucleotides targeting miR-21 have been proposed as therapeutic agents to restore the regulatory balance in tumor cells [17,18,19]. However, effective delivery remains a major challenge.

A previous study examined the effects of pristine graphene oxide (GO) and GO functionalized with antisense miR-21 (GO-antisense miR-21) on HepG2 hepatocarcinoma cells. Exposure to pristine-GO resulted in significant upregulation of genes associated with oncogenic pathways and inflammatory responses, including ICAM-1, TIMP-1, IL-8, MCP-1, and NF-κB. These findings indicate a potential pro-inflammatory and pro-tumorigenic effect of unmodified GO. In contrast, functionalization of GO with antisense miR-21 reversed this pattern, resulting in downregulation of the same genes and suggesting an anti-inflammatory and antitumor effect mediated by miR-21 silencing [20].

To validate these promising in vitro findings and explore the translational potential of this nanosystem, we extended our investigation to an *in ovo* model. We employed the chorioallantoic membrane (CAM) of the chicken embryo—an established and ethically accepted platform for studying tumor growth, angiogenesis, and drug delivery in a cost-effective and highly vascularized setting [21,22]. The CAM tumor model enables the real-time assessment of human tumor development and vascular response, providing an ideal environment for testing anti-angiogenic and antitumoral therapies [23,24].

The goal of this study was to evaluate the therapeutic potential of a GO-based nanosystem functionalized with antisense miR-21 in a translational *in ovo* model. HepG2-derived tumors established on the chorioallantoic membrane (CAM) were treated with either unmodified GO or the GO–antisense miR-21 complex. To assess the efficacy of these treatments, we examined changes in tumor volume, vascular architecture, and the expression of key genes previously analyzed in vitro, including *IL-8*, *MCP-1*, *TIMP-2*, *ICAM-1*, and *NF*-*κB*. Notably, the pro-angiogenic cytokine IL-8 exhibited marked regulation *in ovo*, prompting further protein-level analysis using immunofluorescence to correlate transcriptional and translational responses. Additionally, to confirm targeted delivery of the therapeutic complex, we used a FITC-labeled GO formulation and visualized its accumulation in tumor tissue using confocal microscopy. Altogether, this study provides further evidence of the therapeutic potential of the GO-antisense miRNA 21 nanosystem, demonstrating its ability to suppress tumor growth and angiogenesis by modulating key pro-inflammatory and oncogenic pathways. Indeed, we confirmed that conjugating GO with a miR-21 antisense oligonucleotide successfully counteracts its inherent pro-inflammatory properties. This modification simultaneously transforms the nanomaterial into an effective delivery system that drives antitumor and anti-angiogenic activities *in ovo*. These findings support the development of multifunctional, gene-regulating nanocarriers as innovative tools for treating hepatocellular carcinoma.

## 2. Materials and Methods

### 2.1. Synthesis, Characterization, and FITC Labeling of the GO–Antisense miR-21 Complex

Graphene oxide (GO) and antisense miR-21 oligonucleotides, as well as the GO–miRNA complex, were prepared and characterized as previously described in our earlier study [20]. Transmission electron microscopy (TEM/STEM) analyses revealed wrinkled GO flakes and a homogeneous distribution of antisense miR-21 on the GO surface without detectable aggregation. Fourier-transform infrared (FTIR) spectra confirmed the presence of carbonyl and graphitic groups in GO, as well as characteristic amide and phosphate bands in miRNA. The GO–miRNA complex retained the main spectral features of GO, with a slight decrease in the 3200–3500 cm^−1^ region and the appearance of additional bands in the 1500–1700 cm^−1^ range, indicating successful interaction between the components. Dynamic light scattering (DLS) measurements indicated hydrodynamic diameters of approximately 800 nm for GO, 150 nm for miRNA, and 950 nm for the GO–miRNA complex, accompanied by a shift in ζ-potential from −26.1 ± 7.43 mV to −18.7 ± 8.51 mV, consistent with surface functionalization. In 10% fetal bovine serum (FBS), the nanosystem maintained a submicrometer size distribution without the formation of large aggregates and exhibited a slightly less negative ζ-potential, suggesting good colloidal stability under physiological conditions. UV–vis spectroscopy revealed characteristic absorption bands for both GO and miRNA, with broadening of the bands upon complex formation. Laser-induced breakdown spectroscopy (LIBS) analysis detected carbon emissions in all samples and a nitrogen signal exclusively in miRNA-containing systems, further confirming successful loading. Release studies conducted at pH 4.5 and 7.4 demonstrated faster and more sustained release under acidic conditions, whereas at physiological pH (7.4), a release peak at 24 h was followed by a decline. Kinetic modeling revealed higher Higuchi constants at pH 4.5 (0.030–0.045 vs. 0.015–0.025), while Korsmeyer–Peppas exponents (*n* = 0.32–0.41) indicated a Fickian diffusion mechanism. The Weibull model provided the best fit to the experimental data (R^2^ = 0.96–0.99; β = 0.55–0.75). Enhanced release at acidic pH was further supported by higher initial release rates, reduced t_50_ values (−30–45%), and t_90_ being reached within 48–60 h only under acidic conditions. To facilitate confocal microscopy visualization, the nanosystems were labeled with fluorescein isothiocyanate (FITC) via non-covalent adsorption. Briefly, FITC was added to GO or GO–antisense miR-21 dispersions and incubated overnight at 4 °C in the dark under gentle agitation. This labeling strategy exploits the high surface area and stacking capabilities of graphene oxide for stable dye loading. Successful labeling and probe stability were confirmed by flow cytometry and confocal microscopy, as previously validated, ensuring that the fluorescent signals accurately reflect the intracellular localization of the functionalized nanocarriers.

### 2.2. Culture of HepG2 on Chorioallantoic Membrane

Ethics statement: The Ethics review board II Local Committee for Ethics in Animal Research of Warsaw University of Life Sciences—SGGW considers that this type of project does not fall under the legislation for the protection of animals used for scientific purposes (national decree-law Dz. U. 2015 poz. 266 and 2010–63-EU directive). Fertilized chicken eggs (Gallus gallus, *n* = 24) obtained from a commercial supplier were incubated under standard conditions (37 °C, 60% humidity) for 6 days. Next, a silicone ring containing 7 × 10^5^ HepG2 cells suspended in 50 μL of culture medium was placed onto the chorioallantoic membrane, following the procedure described by Grodzik et al. [25]. Embryos were then incubated for an additional 7 days, after which 12 embryos exhibiting visible tumor formation were selected and randomly divided into three experimental groups of 4 embryos: control group, the GO-treated and the GO-Antisense miR-21 nanosystem. Intratumorally administration of 200 μL of GO 10.0 µg/mL: miRNA 5.0 pmol/mL nanosystems was performed under sterile conditions by injection with an insulin syringe (Ø = 29 mm) in a laminar cabinet. Following treatment, embryos were incubated for a further 3 days. This experiment was terminated on day 18 of embryonic development. Embryos were sacrificed, and tumor tissues were carefully resected for subsequent analysis.

### 2.3. Tumor Monitoring and Volume Measurement

Tumor morphology was assessed using a stereomicroscope (SZX10; CellD software, version 3.1; Olympus Corporation, Tokyo, Japan). Tumor diameter measurements were performed using CellSens Dimension Desktop version 1.3 (Olympus). The tumor volume was calculated using the following formula:

V = 4/3 πr3, where r = 1/2 √(diameter1 × diameter 2), π = 3.1415.

Tumor weight and volume were assessed across independent biological replicates for each experimental condition: untreated control (*n* = 3), pristine GO (*n* = 3), and GO–antisense miR-21 complex (*n* = 3). Each individual tumor harvested from the CAM was treated as a distinct biological replicate to ensure statistical robustness.

### 2.4. Total RNA Extraction and Real-Time PCR

Total RNA was extracted using the PureLink^®^ RNA Mini Kit (Ambion™ Life Technologies, Foster City, CA, USA). Approximately 20 mg of tumor tissue was homogenized in lysis buffer supplemented with 1% 2-mercaptoethanol using a TissueLyser ball mill (Qiagen, Germantown, MD, USA) for two cycles of 5 min at 50 Hz. The homogenates were centrifuged at 12,000× *g*, and the resulting pellets were discarded. The supernatant containing total RNA was transferred to a new tube and processed according to the manufacturer’s protocol. RNA was eluted in 50 µL of RNase-free water and stored at −80 °C until further use. The RNA concentration and purity were determined using a NanoDrop 2000 spectrophotometer (Thermo Scientific, Wilmington, DE, USA). Complementary DNA (cDNA) was synthesized from 2200 ng of RNA using the cDNA High-Capacity Reverse Transcription Kit (Applied Biosystems, Foster City, CA, USA), following the manufacturer’s instructions. The concentration of the obtained cDNA was measured using a NanoDrop 2000 spectrophotometer, and samples were stored at −20 °C until further analysis. Gene expression levels were evaluated by quantitative real-time PCR using the ΔΔCt method (ΔΔCt = ΔCt test sample − ΔCt calibrator sample). The qPCR reactions were performed in 48-well plates using Luminaris Color HiGreen qPCR Master Mix (Thermo Fisher Scientific, Waltham, MA, USA), with 100 ng of cDNA for each reaction. The expression of the following genes was analyzed: *IL-8*, *MCP-1*, *TIMP-2*, *NF-Kb* and *ICAM-1*. Primer sequences are listed in Table 1, and *GAPDH* was used as the housekeeping reference gene. Amplification conditions followed the manufacturer’s recommendations, and all samples were analyzed in triplicate using a StepOnePlus™ Real-Time PCR System (Applied Biosystems, Foster City, CA, USA) using StepOne Software v2.3.

### 2.5. Confocal Microscopy

The confocal microscope was used to perform immunofluorescence on tumor tissue and to detect GO-FITC and GO-antisense miR-21 within the tumor. The tumor tissue was sectioned using a Cryostat microtome (CM 1860; Leica Microsystems, Wetzlar, Germany). Tumor tissues treated with GO-conjugated with FITC and GO–Antisense miR-21 conjugated with FITC were placed on glass slides and fixed with 4% paraformaldehyde, and after 20 min, the nuclei were stained with DAPI and the cytoskeleton with Actin Red (R37112 Thermo Scientific, Wilmington, DE, USA). The FITC-positive tumor tissue, observed as green fluorescence, was recorded under a confocal microscope (IX81 FV1000, Olympus Corporation, Tokyo, Japan) with FVIO-ASW ver. 1.7c software (Olympus Corporation, Tokyo, Japan). For immunofluorescence analysis, tumor tissue sections were first fixed with 4% paraformaldehyde (PFA) and subsequently permeabilized with 0.5% Tween 20 for 10 min. The tissues were then blocked in a PBS solution containing 1% BSA and 2% goat serum for 30 min. Following blocking, tissues were treated with a 0.25% glycine solution. After PBS washes, the sections were incubated overnight with the primary antibody, IL-8 (Invitrogen, Carlsbad, CA, USA; AHC0881). The following day, the sections were incubated with the secondary antibody (Goat anti-Rabbit IgG (H + L) Highly Cross-Adsorbed Secondary Antibody, Alexa Fluor™ Plus 555, A32732, Invitrogen, Carlsbad, CA, USA) for 1 h. Finally, nuclei were stained with DAPI, and the tumor tissues were observed under a confocal microscope (IX81 FV1000, Olympus Corporation, Tokyo, Japan) using FVIO-ASW ver. 1.7c software (Olympus Corporation, Tokyo, Japan).

### 2.6. Image Analysis

Image analysis was performed using ImageJ software (NIH, Bethesda, MD, USA) version 1.54. For each image, the region of interest (ROI) corresponding to the CAM tumor area was manually defined. Images were converted to 8-bit grayscale, and a consistent threshold was applied to identify IL-8 positive staining. The percentage of IL-8-positive area was calculated as the ratio of threshold-positive pixels to the total ROI area. The same threshold parameters were applied to all images to ensure consistency.

### 2.7. Statistical Analysis

Data analysis was conducted using GraphPad Prism (version 8). Results are reported as mean ± standard deviation (SD) from independent biological replicates. Statistical differences between experimental groups were assessed by one-way ANOVA followed by Tukey’s post hoc test for multiple comparisons. The number of biological replicates (*n*) is indicated in the corresponding figure and table legends. Significance levels were set at * *p* < 0.05, ** *p* < 0.01, *** *p* < 0.001, and **** *p* < 0.0001, with *p* < 0.05 considered statistically significant.

### 2.8. Use of Generative AI Tools

Generative AI Tools (ChatGPT, OpenAI, GPT-5.3) were used during the preparation of this manuscript for language editing, grammar correction and improvement of text clarity and organization. No AI tools were used for data analysis, data interpretation or generation of scientific results. All outputs were critically reviewed, edited and validated by the authors, who take full responsibility for the content of the manuscript.

## 3. Results

### 3.1. GO-Mediated Delivery of Antisense miR-21 Suppresses Tumor Growth and Angiogenesis

**Figure 1:**
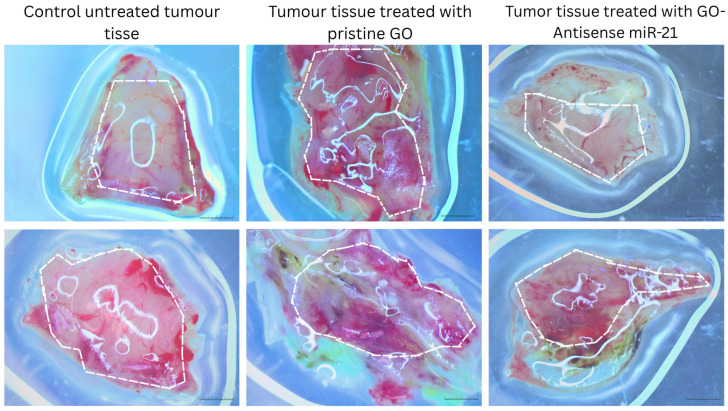
HepG2 tumor tissue, untreated and treated with pristine GO and GO-antisense miRNA 21. Representative duplicate images of HepG2-derived tumors following treatment with pristine GO and GO functionalized with antisense miR-21. The images show typical tumors from each experimental group. White lines delineate the tumor boundaries from the CAM model. Scale bar: 2 mm.

To evaluate the therapeutic effects of the GO-based nanosystem, we examined its impact on tumor growth and blood vessel formation using an *in ovo* model. Specifically, we compared the effects of GO and GO functionalized with antisense miR-21 on tumors derived from HepG2 cells. The goal was to determine whether delivery of the antisense miR-21 could slow tumor progression and angiogenesis. In the CAM tumor model, treatment with GO resulted in greater tumor weight (Table 2) and increased vascularization (Figure 1). However, there is no significant increase in overall tumor volume compared to untreated controls. This suggests tissue expansion associated with remodeling and neovascularization rather than an increase in solid tumor mass. In contrast, treatment with the GO–antisense miRNA-21 nanosystem significantly reduced the tumor mass, volume, and vascular density. These findings indicate that targeted inhibition of miRNA-21 delivered via GO effectively suppresses tumor growth and angiogenesis. Images of four independent tumors for each experimental group are provided in the Supplementary Materials (Figure S1).

### 3.2. Visualization of GO and GO–miR-21 Localization in Tumor Tissue

**Figure 2:**
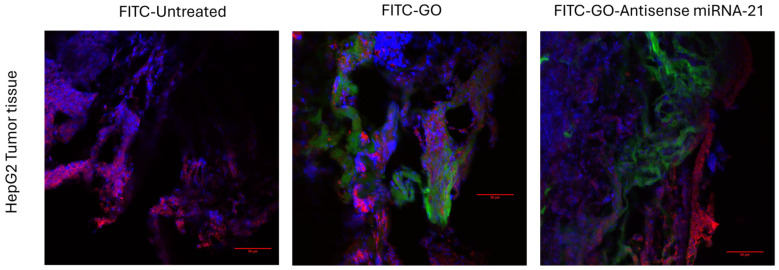
Confocal microscopy analysis of tumor tissue following treatment with FITC-labeled GO and GO–antisense miR-21 nanosystems. Representative images illustrate the intratumoral distribution of FITC-labeled graphene oxide (FITC-GO) and GO functionalized with antisense miR-21. Green fluorescence indicates the presence of the GO-based nanosystems, red marks the cytoskeleton, and blue highlights cell nuclei. Scale bar: 50 µm.

To confirm the tumor-specific localization of the nanosystem, we employed confocal microscopy to visualize FITC-labeled GO in resected tumor tissues (Figure 2). No fluorescence signal was detected in untreated controls, confirming the specificity of FITC labeling. In contrast, green fluorescence was clearly observed in tumors treated with either FITC-GO or FITC–GO–antisense miR-21, indicating successful accumulation of the nanomaterial within the tumor mass. Notably, FITC-GO exhibited a diffuse distribution throughout the tumor tissue, whereas FITC–GO–antisense miR-21 was predominantly localized at the tumor margins, avoiding acellular regions. These findings confirm the presence of GO-based nanosystems in tumor tissue and reveal distinct distribution patterns depending on the functionalization.

### 3.3. Gene Expression Modulation by GO and GO–Antisense miR-21 Nanosystem in HepG2 Tumor Tissue

**Figure 3:**
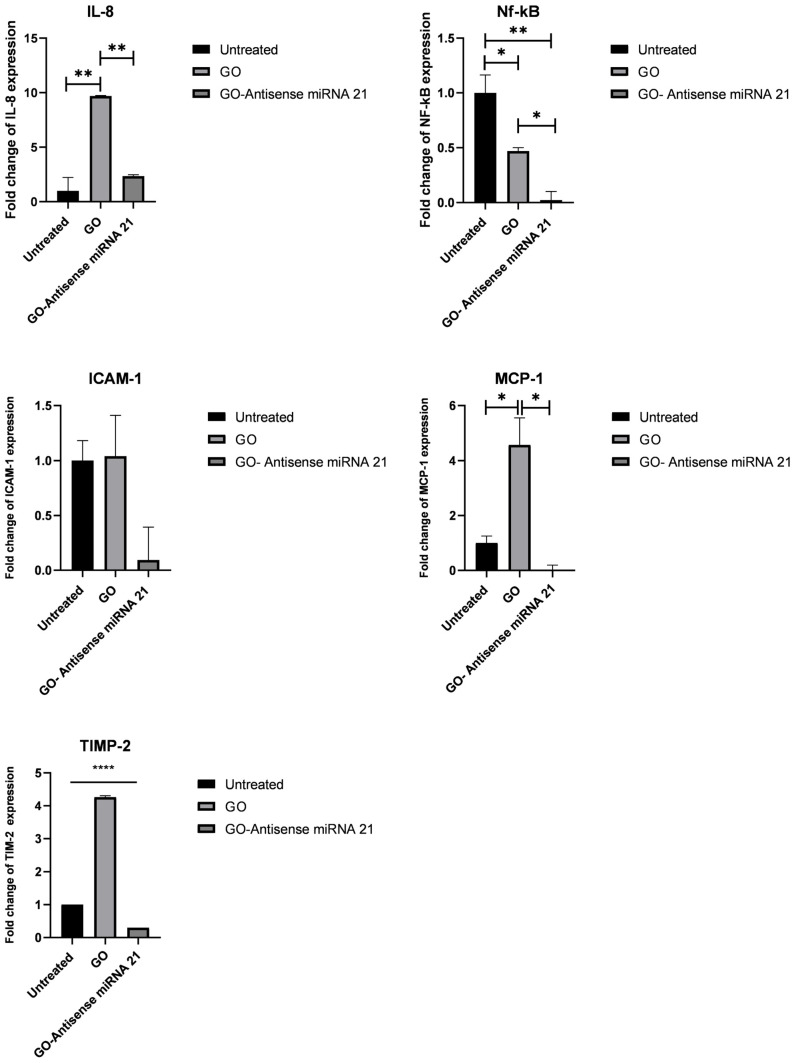
Transcriptional levels of IL-8, MCP-1, ICAM-1, TIMP-2 and NF-κB in HepG2-derived CAM tumor tissues. Tumors were untreated or treated with pristine graphene oxide (GO) and GO–antisense miR-21 nanosystem. After treatment, tumor tissues were collected, and total RNA was extracted. Gene expression levels were determined by qPCR and calculated using the 2^^−ΔΔCt^ method. Data are presented as mean ± SD from independent biological replicates and expressed as fold change relative to the housekeeping gene. Statistical analysis was performed using one-way ANOVA followed by Tukey’s multiple comparison test. Significance levels are indicated as follows: **** *p* < 0.0001; ** *p* < 0.01; * *p* < 0.05.

To further investigate the biological effects of the treatments observed *in ovo*, the expression of key genes involved in inflammation and tumor progression was analyzed in HepG2-derived tumor tissues. Specifically, the genes examined were *IL-8*, *MCP-1*, *TIMP-2*, *ICAM-1*, and *NF-κB*. By assessing these gene expression profiles, we could evaluate how GO and the GO–antisense miR-21 nanosystem influence molecular pathways underlying their distinct effects on tumor growth and vascularization. Notably, in samples treated with GO, significant upregulation of the pro-inflammatory genes *IL-8* and *MCP-1* was observed. In addition, increased expression of *TIMP-2* and *ICAM-1*, associated with extracellular matrix remodeling and cell adhesion, was observed. In contrast, *NF-κB* expression was lower than in the control group. This suggests a possible compensatory mechanism or selective modulation of inflammatory pathways. Interestingly, samples treated with the functionalized GO–antisense miR-21 showed a significant downregulation of all the analyzed genes (Figure 3). This indicates an anti-inflammatory effect and a potential reduction in the pro-tumoral response induced by treatment with non-functionalized GO. Overall, these results highlight a key role of the nanocomplex in selectively modulating gene expression related to inflammation and tumor progression. The results also align with the effects previously observed in vitro [20].

### 3.4. Immunofluorescence Analysis of IL-8 Expression in HepG2-Derived Tumor Tissues

**Figure 4:**
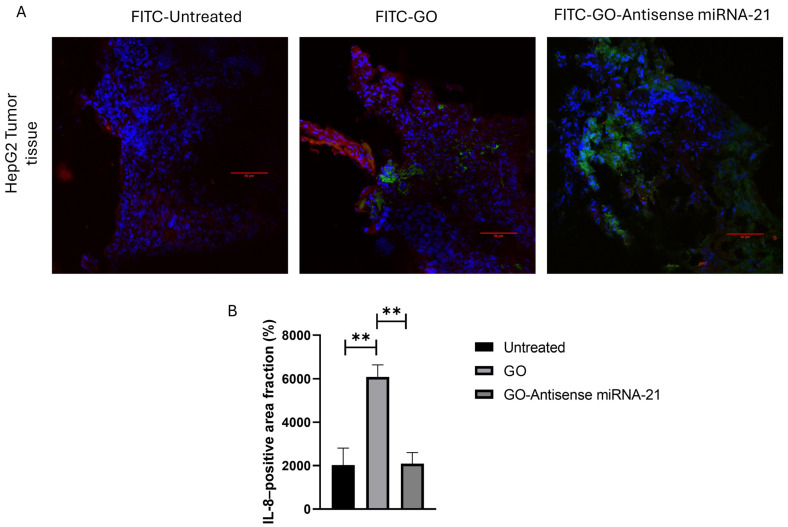
Immunofluorescence analysis of IL-8 protein expression in HepG2 tumor tissues following treatment with FITC-GO and FITC–GO–antisense miR-21 nanosystems. (**A**) Representative confocal images of untreated control tumors (left), GO-treated tumors (middle), and GO–antisense miR-21–treated tumors (right). Nuclei are counterstained with DAPI (blue), IL-8 is shown in red, and FITC-labeled GO is shown in green. Scale bars: 50 μm. (**B**) Quantification of IL-8-positive area (percentage of total field) based on ImageJ analysis. Data are presented as mean ± SD (*n* = 4 tumors per group; 3 fields per tumor). Statistical analysis was performed using one-way ANOVA followed by Tukey’s post hoc test; ** *p* < 0.01.

Among the genes analyzed, IL-8 was selected for protein expression validation because it showed a pronounced modulation not only in the present *in ovo* CAM tumor model but also consistently in our previous in vitro study [20]. This consistency highlighted IL-8 as an appropriate marker to verify whether transcriptional regulation was also reflected at the protein level within the tumor microenvironment. To further validate the gene expression results at the protein level, immunofluorescence staining was performed to assess IL-8 expression in HepG2-derived tumor tissues. Given the observed modulation of IL-8 at the transcript level, this analysis aimed to visualize how the GO and GO–antisense miR-21 nanosystem affects the distribution of IL-8 protein within the tumor microenvironment. Tissue sections from untreated tumors (control), tumors treated with FITC-GO, and tumors treated with FITC-GO-antisense-miRNA21 were stained using an anti-IL-8 antibody and appropriate fluorescent secondary antibody. Nuclei were counterstained with DAPI to visualize the tissue architecture, and FITC labeling confirmed the presence of the nanomaterials. Figure 4A demonstrates that untreated control tissues exhibited a moderate IL-8 signal, indicating basal IL-8 expression in the tumor. Tumors treated with FITC-GO displayed a substantial increase in IL-8 fluorescence, characterized by a widespread, intense signal throughout the tissue. In contrast, tissues treated with the FITC-GO-antisense-miRNA21 nanosystem exhibited only a few discrete IL-8-positive spots, indicating lower IL-8 protein levels compared to FITC-GO treatment. These results highlight differential modulation of IL-8 protein expression by the treatments, consistent with transcriptional changes observed in qRT-PCR. These qualitative observations were confirmed by the quantitative analysis shown in Figure 4B, which showed that the percentage of IL-8-positive area was significantly higher in GO-treated tumors than in controls and markedly lower in tumors treated with the GO–antisense miR-21 nanosystem.

## 4. Discussion

Gene therapy has traditionally relied on viral vectors due to their high efficiency in delivering nucleic acids and achieving sustained gene expression [26]. However, despite their effectiveness, viral systems present important limitations, including risks of insertional mutagenesis and immunogenicity, limited cargo capacity, and complex manufacturing processes. These challenges have restricted their broader clinical application, particularly in complex diseases such as cancer. As a result, increasing attention has shifted toward non-viral delivery platforms, including lipid nanoparticles, polymeric carriers, and carbon-based nanomaterials. Among these, GO has recently emerged as a promising and versatile carrier for nucleic acid delivery due to its favorable physicochemical properties, ease of functionalization, and potential for targeted therapeutic applications [27]. In this context, the findings of this study demonstrate that a GO-based nanosystem functionalized with antisense miR-21 modulates tumor growth, vascularization, and inflammatory signaling in the chorioallantois membrane model. miR-21 is one of the most frequently upregulated oncogenic microRNAs in solid tumors, including hepatocellular carcinoma, where it promotes tumor progression by repressing tumor suppressor genes and activating pro-survival and inflammatory pathways. As a pleiotropic oncomiR, miR-21 orchestrates a vast signaling network by regulating multiple target genes involved in tumor progression. These include key tumor suppressors such as Phosphatase and Tensin Homolog (*PTEN*) and Programmed Cell Death 4 (*PDCD4*), alongside transcription factors like *TFDP3* and *HBP1* (HMG-Box Transcription Factor 1). Furthermore, miR-21 modulates the TGF-β/SMAD7 and HIF-1 alpha pathways, which are critical for epithelial–mesenchymal transition and hypoxia-induced angiogenesis. Its influence also extends to metabolic and inflammatory mediators, including *FABP7*, *IL-12*, and the extracellular matrix regulator *TIMP-3* [28]. miR-21 directly targets *TIMP-3*, and its broader influence on the tumor microenvironment also affects the expression of related factors, thereby driving cell proliferation, invasion, and angiogenesis [28]. Consequently, therapeutic inhibition of miR-21 has been widely explored as a strategy to attenuate tumor growth and metastatic potential. Recently, several studies have explored various approaches to miR-21 inhibition, including antisense oligonucleotides, locked nucleic acids, and nanoparticle-mediated delivery, each exhibiting distinct efficacy profiles and translational barriers [29]. For example, co-delivery of antisense miR-21, with gemcitabine in anti-CD44v6 antibody-coated nanoparticles, enhanced apoptosis and reduced metastasis in pancreatic cancer models [30]. In glioblastoma cells, GO–antisense complexes promoted apoptosis by upregulating *BAX*, *p53*, and *p21*, while suppressing *Bcl-2* and *PCNA*, thereby activating intrinsic and extrinsic apoptotic pathways [31]. GO and its hybrid derivatives have emerged as promising non-viral carriers due to their high loading capacity, protection of nucleic acids from degradation, and potential for surface functionalization to achieve targeted delivery [27,32]. Notably, GO-based platforms have demonstrated the ability to co-deliver therapeutic oligonucleotides and anticancer drugs, as well as to incorporate imaging modalities for theranostic applications [33], anticancer drugs, and imaging modalities for theranostic applications [33,34]. Compared to other nanomaterials, such as lipid nanoparticles or polymeric carriers, GO offers a unique balance between structural versatility and multifunctionality. However, the standardization of characterization protocols and long-term safety assessment remain challenges for clinical translation [35,36]. Importantly, our previous study [20] demonstrated that the GO–antisense miR-21 complex was associated with coordinated downregulation of oncogenic and pro-inflammatory pathways linked to miR-21 signaling, including *IL-8*, *MCP-1*, *TIMP-2*, *ICAM-1*, and *NF-κB*. Building on these findings, the present study further characterizes how functionalization modulates this baseline response in a more complex CAM tumor model. Our findings, obtained using the CAM tumor model, demonstrated that this functionalized nanosystem effectively inhibits tumor growth and angiogenesis. Indeed, in this context, while non-functionalized GO promoted vascular remodeling and inflammatory responses in the tumor microenvironment, functionalization with antisense miR-21 reversed these effects, resulting in a reduced tumor size and vascular density (Figure 1). Interestingly, although GO treatment increased the tumor weight and vascular density, the tumor volume did not increase significantly (Table 2), suggesting that GO may primarily influence stromal remodeling and tumor microenvironment dynamics rather than directly stimulating tumor cell proliferation. Indeed, previous studies have reported that graphene oxide can interact with immune and stromal cells, thereby modulating inflammatory signaling and angiogenic processes within the tumor microenvironment [37,38]. In particular, low-dose exposure to graphene oxide has been reported to cause morphological changes in cancer cell membranes and promote epithelial–mesenchymal transition through activation of the TGF-β signaling pathway, thereby activating downstream transcriptional genes associated with cell migration and metastatic potential [39]. Similarly, in our CAM tumor model, treatment with GO alone caused the upregulation of *IL-8*, *MCP-1*, and *TIMP-2*, indicating a pro-inflammatory and pro-angiogenic response (Figure 3). Otherwise, we observed a decrease in *NF-κB* expression in GO-treated tumors despite simultaneous increases in *IL-8* and *MCP-1*. This suggests that GO may activate inflammatory signals through NF-κB-independent pathways [40].

Indeed, in vivo, inflammatory gene expression is regulated by the combined activity of several transcriptional programs, including MAPK/AP-1 and STAT3 pathways, as well as hypoxia-responsive HIF-1α signaling. *IL-8* and *MCP-1* transcription have been shown to be controlled not only by *NF-κB* but also by *AP-1* and *STAT3* activation and can be further enhanced under hypoxic conditions typical of highly vascularized tumor microenvironments [41,42,43]. Furthermore, the CAM tumor model includes diverse cell populations, such as endothelial, stromal, and immune-related cells, which actively participate in inflammatory signaling and may compensate for reduced *NF-κB* activity in tumor cells [44,45]. Graphene oxide has been reported to induce oxidative stress and activate MAPK pathways, providing alternative routes for cytokine induction independent of *NF-κB* [46,47]. Therefore, the divergence between *NF-κB* expression and cytokine upregulation likely reflects the complexity of tumor microenvironmental regulation and the engagement of compensatory inflammatory circuits. Interestingly, exposure of a CAM tumor to GO did not increase *ICAM-1* transcription levels, a key regulator of leukocyte adhesion and intercellular interactions that typically depend on a fully developed immune system. This finding may reflect an intrinsic limitation of the CAM tumor model, in which the embryonic environment is characterized by an immature immune compartment [48]. As a result, leukocyte-dependent adhesion signaling is underrepresented, which may explain the absence of detectable *ICAM-1* induction.

However, when GO was functionalized with an antisense miR-21, the genes previously upregulated by GO alone became downregulated (Figure 3). Accordingly, tumors treated with the GO-antisense miR-21 nanosystem exhibited a significant reduction in both tumor weight and vascular density (Figure 1). This effect likely reflects the inhibition of miR-21 activity, thereby suppressing oncogenic pathways involved in inflammation, extracellular matrix remodeling, and angiogenesis, which are known to be regulated by miR-21. Immunofluorescence analysis of IL-8 confirmed the transcriptional findings at the protein level (Figure 4). Untreated tumors displayed moderate basal IL-8 expression. This aligns with the typical inflammatory tumor microenvironment [49,50,51].

GO treatment substantially increased the IL-8-positive area fraction. This result confirms the induction of an inflammatory response and matches the higher fold change observed in GO-treated tumors (Figure 4). In contrast, tumors treated with the GO–antisense miR-21 nanosystem showed significantly reduced protein expression. Only a few discrete positive spots were seen. This result supports the hypothesis that GO functionalization with the antisense oligonucleotide not only mitigates the pro-inflammatory effect of GO but also actively suppresses tumor-associated IL-8-mediated inflammation. Confocal microscopy demonstrated that both nanosystems were internalized by cells. The functionalized complex accumulated mainly at cellular interfaces and in regions of higher cell density. This is likely due to local diffusion and tissue distribution after injection. This distinct localization pattern indicates that functionalization of antisense oligonucleotides may influence the nanosystem interactions with the cellular microenvironment, thereby enhancing its biological activity.

In summary, while these results provide promising preliminary evidence, several inherent limitations remain. The use of the HepG2 cancer cell line and a simplified *in ovo* CAM model indicates that these findings represent a robust proof of concept rather than definitive preclinical validation. The intrinsic biological effects of GO, including pro-inflammatory and immunomodulatory responses, remain an important consideration in the design of graphene-based therapeutic platforms. Importantly, the demonstrated ability of this platform to reduce the intrinsic pro-inflammatory effects of GO through targeted miRNA inhibition represents a significant advance toward developing multifunctional, non-viral theranostic platforms.

Our results provide preliminary evidence that this nanosystem may serve as a candidate for tumor growth modulation. The anti-inflammatory and anti-angiogenic effects observed in the CAM tumor model are particularly promising but should be interpreted as preliminary in vivo evidence. Indeed, although the CAM tumor model provides a rapid and cost-effective in vivo platform to investigate tumor growth, angiogenesis, and therapeutic responses, it does not fully capture the systemic complexity or long-term biocompatibility necessary for translation to human applications. Despite these limitations, the CAM assay remains a valuable, ethical, and cost-effective bridge between in vitro screening and mammalian in vivo models, enabling rapid assessment of anti-angiogenic properties.

## 5. Conclusions

Graphene oxide is a promising nanocarrier for nucleic-acid-based therapeutics due to its high loading capacity and physicochemical versatility. However, unmodified GO can induce pro-inflammatory and tumor-modulating effects in the tumor microenvironment. Our findings demonstrate that functionalization of graphene oxide with antisense miR-21 shifts its biological effects within the tumor microenvironment from a pro-inflammatory and pro-angiogenic profile toward a suppressive one. These findings underscore the potential of integrating nanomaterial platforms with microRNA-based strategies to achieve more precise and context-specific therapeutic outcomes. While the CAM tumor model provides a rapid and informative system for investigating tumor growth and angiogenesis, its inherent limitations necessitate additional validation in mammalian models to evaluate long-term efficacy, safety, and translational relevance. Subsequent research should prioritize improving the interfacial affinity of the nanosystem to enhance localization and assess its applicability in targeting additional oncogenic biomarkers. This study paves the way for translating molecular antisense strategies into effective, cost-efficient, and rapid therapeutic tools.

## Figures and Tables

**Table 1:** Primer sequences. Abbreviations: *ICAM-1*: intercellular adhesion molecule 1, *MCP-1*: monocyte chemoattractant protein-1, *TIMP-2*: tissue inhibitor of metalloproteinases 2, *IL-8*: interleukin 8, *NF-kB*: nuclear factor kappa-B, *GAPDH*: glyceraldehyde 3-phosphate dehydrogenase.

Target Gene	Forward Primer	Reverse Primer
*ICAM-1*	AGCGGCTGACGTGTGCAGTAAT	TCTGAGACCTCTGGCTTCGTCA
*MCP-1*	CCACGCAACAAATGAAGTAGCCC	CTGGAATGCTGTTCCCTTCAAG
*TIMP-2*	ACCCTCTGTGACTTCATCGTGC	GGAGATGTAGCACGGGATCATG
*IL-8*	GAGAGTGATTGAGAGTGGACCAC	CACAACCCTCTGCACCCAGTTT
*NF-kB*	GGCAGACCAGTGTCATTGAGCA	CAGCAGAAAGCTCACCACACTC
*GAPDH*	TGCACCACCAACTGCTTAGC	GGCATGGACTGTGGTCATGAG

**Table 2:** Mass and volume of HepG2 tumor tissue, untreated and treated with GO and GO-antisense miRNA 21. Tumor mass and volume were measured post-treatment and compared to untreated controls. Quantitative data were obtained from independent tumors (control: *n* = 3; GO: *n* = 3; GO–antisense miR-21: *n* = 3), and values are reported as mean ± SD. **** *p* < 0.0001; *** *p* < 0.001; ** *p* < 0.01.

**Condition**	**Tumor Weight (mg)**	**Tumor Volume (cm^3^)**
Untreated tumor	97.88 ± 14.9	158.16 ± 12.66
Tumor treated with GO	186.7 ± 16.67 ***	90.68 ± 24.2 **
Tumor treated with GO-antisense miR-21	45.5 ± 13.22 ****	20.72 ± 12.32 **

## Data Availability

The original contributions presented in this study are included in the article/Supplementary Material. Further inquiries can be directed to the corresponding author(s).

## Supplementary Materials

The following supporting information can be downloaded at https://www.mdpi.com/article/10.3390/biom16040523/s1, Figure S1: Representative images of HepG2-derived CAM tumors, untreated or treated with pristine GO and GO functionalized with antisense miR-21. Four independent tumor samples per group are shown. White lines delineate the tumor boundaries from the CAM model. Scale bar: 2 mm.

## Abbreviations

The following abbreviations are used in this manuscript:

AP-1	Activator protein-1
CAM	Chick embryo chorioallantoic membrane
cDNA	Complementary DNA
DAPI	4′,6-diamidino-2-phenylindole
FITC	Fluorescein isothiocyanate
GAPDH	Glyceraldehyde 3-phosphate dehydrogenase
GO	Graphene oxide
HCC	Hepatocellular carcinoma
HIF-1 α	Hypoxia-inducible factor-1 alpha
ICAM-1	Intercellular adhesion molecule 1
IL-8	Interleukin 8
MAPK	Mitogen-activated protein kinase
MCP-1	Monocyte chemoattractant protein-1
miR-21	MicroRNA-21
NF-kB	Nuclear factor kappa-B
PBS	Phosphate-buffered saline
PFA	Paraformaldehyde
STAT3	Signal transducer and activator of transcription 3
TIMP-2	Tissue inhibitor of metalloproteinases 2
