# Supplementary material for: Graphene Oxide–Antisense miR-21 Nanosystem Modulates Gene Expression and Suppresses Tumorigenesis in HepG2-Derived CAM Xenografts
Source: Biomolecules. 2026 Apr 1;16(4):523. doi: 10.3390/biom16040523 (PMC13113956; doi:10.3390/biom16040523)
Supplement: Supplementary file 1 [file biomolecules-16-00523-s001.zip › biomolecules-4177576-supplementary.pdf]

biomolecules

Supplementary information

Graphene Oxide–Antisense miR-21 nanosystem modulates gene expression and suppresses tumorigenesis in HepG2-derived CAM xenografts

Paola Trischitta [1,2], Barbara Nasiłowska [3], Rosamaria Pennisi [1], Marianna Costa [1,2], Maria Teresa Sciortino [1,*] and Marta Kutwin [4,*]

[1] Department of Chemical, Biological, Pharmaceutical and Environmental Science, University of Messina, Viale Ferdinando Stagno d'Alcontres 31, 98166 Messina, Italy. rpennisi@unime.it, maria.sciortino@unime.it
[2] Department of Chemistry, Biology, and Biotechnology, University of Perugia, Via Elce di Sotto 8, 06123 Perugia, Italy. paola.trischitta@dottorandi.unipg.it; marianna.costa1@studenti.unime.it
[3] Institute of Optoelectronics, Military University of Technology, Gen. S. Kaliskiego 2, 00-908 Warsaw, Poland. barbara.nasilowska@wat.edu.pl
[4] Department of Nanobiotechnology, Institute of Biology, Warsaw University of Life Sciences, Ciszewskiego 8, 02-786 Warsaw, Poland marta_kutwin@sggw.edu.pl
* Correspondence: mtsciortino@unime.it (M.T.S); marta_kutwin@sggw.edu.pl. (M.K.)

Academic Editor: Rita Cortesi, Maddalena Sguizzato and Francesca Ferrara

Received: 11 February 2026
Revised: 24 March 2026
Accepted: 27 March 2026
Published: 1 April 2026

Copyright: © 2026 by the authors. Submitted for possible open access publication under the terms and conditions of the Creative Commons Attribution (CC BY) license.

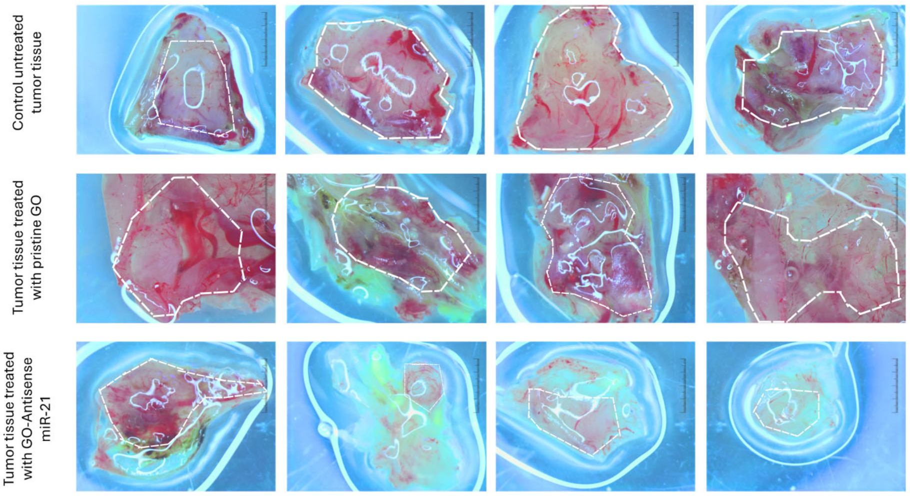

Figure S1. Representative images of HepG2-derived CAM tumors, untreated or treated with pristine GO and GO functionalized with antisense miR-21. Four independent tumor samples per group are shown. White lines delineate the tumor boundaries from the CAM model. Scale bar: 2 mm.
